# Work conditions and determinants of health status among industrial shift workers: a cross-sectional study

**DOI:** 10.3389/fpubh.2024.1489178

**Published:** 2025-01-07

**Authors:** Sasha Javanmardi, Ludwig Rappelt, Christian Baumgart, Daniel Niederer, Lars Heinke, Jürgen Freiwald

**Affiliations:** ^1^Department of Movement and Training Science, University of Wuppertal, Wuppertal, Germany; ^2^Department of Intervention Research in Exercise Training, German Sport University Cologne, Cologne, Germany; ^3^Department of Sports Medicine and Exercise Physiology, Institute of Occupational, Social and Environmental Medicine, Goethe University Frankfurt, Frankfurt/Main, Germany

**Keywords:** workplace health promotion, occupational health, occupational hazards, musculoskeletal disorders, job-profile

## Abstract

**Introduction:**

This study investigated potential health status differences among forging, manufacturing, and logistics workers.

**Methods:**

We included 403 participants (age: 41 ± 12 years) from a medium-sized steel company (forge: 64, manufacturing: 299, logistics: 99). Health status was multifactorial assessed: (1) Frequency of musculoskeletal complaints (German Pain Questionnaire). (2) Pain intensity, physical and psychological load [visual analog scales (VAS) 0–100 points]. (3) Occupational moderate-to-vigorous physical activity (MVPA), total MVPA, and sedentary behavior [Global Physical Activity Questionnaire (GPAQ)]. (4) Quality of life [Short Form Health Survey (SF-36)]. Between-group effects were analyzed via one-way ANOVAs with post-hoc Tukey correction.

**Results:**

308 workers (76.4%) reported at least one musculoskeletal issue. A significant between-group difference was revealed for left shoulder [*F*(2,40) = 5.40; *p* = 0.008; ω^2^ = 0.17], occupational MVPA [*F*(2,368) = 9.49; *p* < 0.001; ω^2^ = 0.04] and total MVPA [*F*(2,368) = 6.90; *p* = 0.001; ω^2^ = 0.03]. Post-hoc tests revealed a difference (*p* ≤ 0.007) between manufacturing (left shoulder: *n* = 22; 42.5 ± 24.8; occupational MVPA: *n* = 219; 6,978 ± 5,137 METs min/week; total MVPA: *n* = 219; 8,471 ± 5,390 METs min/week) and logistics workers (left shoulder: *n* = 14; 70.4 ± 26.3 au; occupational MVPA: *n* = 96; 9,640 ± 4,605 METs min/week; total MVPA: *n* = 96; 10,856 ± 4,680 METs min/week). No other between-group differences were observed.

**Discussion:**

Variations in health disparities across work conditions were observed. Yet, clear distinctions between work conditions and health outcomes remain a challenge. Effective interventions should be focused on job-specific and personalized health profiles rather than a stratification of work conditions to enhance health, productivity, and workforce sustainability.

## 1 Introduction

Industrial work is recognized as one of the most physically demanding and mentally challenging occupational sectors (1). Beyond the physical demands, workers are frequently exposed to various occupational hazards, including dust, noise, vibration, awkward postures, repetitive movements, high-force exertion, and high impacts (1–4). These demands affect workers’ health, increasing the risk of illnesses, injuries, and chronic diseases (5, 6). Among these, work-related musculoskeletal disorders remain a major global concern among industrial workers, characterized by high prevalence rates and a tendency for persistent, long-term complaints despite low incidence ratios (7–9). For instance, in 2019, more than 50% of manufacturing workers in the EU reported absences due to work-related musculoskeletal disorders, exceeding those caused by flu-related absences (8, 10). Regarding body zones, the back is the most frequently affected body region, followed by the shoulder/neck, wrist, and knee, underscoring the widespread burden across multiple body regions in this population (7–9).

Addressing these challenges requires a proactive approach to workplace health promotion, which has been shown to enhance worker health and productivity by targeting factors that influence well-being (11, 12). For instance, the early detection of external factors influencing health status is crucial for timely diagnosis and preventive care, leading to long-term benefits (13). According to the International Labor Organization (ILO), global trends such as globalization, technological advancements, demographic shifts, and climate change are reshaping the nature of work (14), further emphasizing the importance of workplaces as a platform for promoting healthy habits from a public health perspective to address these challenges (11).

Despite these efforts, standardized health promotion programs often fail to address the complexity of the industrial work environments. Previous research suggests that a one-size-fits-all approach may be too simplistic, and health interventions should consider the diverse working conditions and health disparities within industrial sectors (15–17). A distinction based on working conditions may be a viable approach for assessing health-related factors. This cross-sectional study aimed to determine whether different working conditions among industrial workers influence health outcomes. These findings intend to guide stakeholders in developing tailored promotion strategies to address the specific needs of the workforce (18).

## 2 Materials and methods

### 2.1 Design and ethics

This cross-sectional study investigated industrial workers of a medium-sized steel company in Germany. The local ethics committee approved the study, including all described procedures (SK/AE240527). Before starting data collection, all participants were informed of the study procedure and aim. Then, they voluntarily signed a written informed consent form.

### 2.2 Population and setting

Data collection for this study was conducted between April 2022 and March 2023 by a team of trained research students led by experienced investigators. The team visited the company and screened potentially eligible workers, independent of any company representatives. None of the team members had any personal relationship with the participants.

The inclusion criteria for this study were: (I) age between 18 and 65 years, and (II) current full-time employment as a rotating shift worker in one of three working conditions. Work conditions are physical, environmental, and organizational factors specific to each department, reflecting the cumulative demands and exposure characteristics. The company was stratified into three conditions: forging (high physical and environmental stress, such as heat and noise), manufacturing (moderate physical demands with repetitive tasks), and logistics (dynamic physical activities like lifting and transporting). Participants were excluded if they worked across multiple conditions or were employed as temporary workers.

A total of 1.116 industrial workers were invited to participate in this study. Within the described company’s stratum, 206 workers were engaged in forging, 577 in manufacturing, and 333 in logistics.

### 2.3 Procedure

An initial interview was followed by a survey. The procedure encompassed five domains: demographic/anthropometric information, orthopedic complaints, physical activity, quality of life, and assessment of physical and psychological load. Subsequently, all participants provided a paper-and-pencil-based version of the surveys presented in German.

### 2.4 Measurements and outcomes

#### 2.4.1 Pain frequency and intensity

A Part of the German Pain Questionnaire, a validated and reliable tool for assessing musculoskeletal complaints (19), was used to evaluate the location and frequency of orthopedic issues. Participants were presented with a body diagram and instructed to circle any anatomical regions where they experienced pain. The reported pain locations were sorted into the following regions: neck, upper back, right/left shoulder, right/left elbow, right/left wrist, right/left hand, lower back, hip, right/left knee, and foot. Pain frequency was quantified by counting the total number of anatomical regions with reported pain, providing a cumulative measure of musculoskeletal burden for each participant. In addition, pain intensity of each region was graded using a visual analog scale (VAS), ranging from 0 to 10 cm at regular intervals. The VAS is a recognized and reliable tool for measuring pain intensity (20).

#### 2.4.2 Physical activity

The Global Physical Activity Questionnaire (GPAQ) is one of the World Health Organisation’s (WHO) stepwise approaches to surveillance of non-communicable disease factors that assess physical activity levels using 16 questions (21). The questionnaire can calculate the overall physical activity levels by assessing each domain’s contribution to overall physical activity (22). Total moderate-to-vigorous physical activity (MVPA) was calculated for occupational and total day as Metabolic Equivalent (METs) minutes per week. Therefore, when calculating METs using GPAQ data, moderate activity equals 4 METs and 8 METs to the time spent on vigorous activity (23); additionally, one extra item collected information about the amount of time spent on sedentary behavior (24). The GPAQ is a suitable and acceptable instrument for monitoring physical activity, and its validity and reliability have been assessed in several countries (24).

#### 2.4.3 Quality of life

The Short Form Health Survey 36 (SF-36) is a questionnaire with 36 items that measure health-related quality of life on eight scales. Principal component analysis revealed two dimensions: the physical dimension represented by the Physical Component Summary (PCS) and the mental dimension represented by the Mental Component Summary (MSC) (25). The scores ranged from 0 to 100, with 0 being the worst and 100 being the best health status (26). The German version of the survey is reliable and valid (27).

#### 2.4.4 Physical and psychological load

Subjective physical and psychological loads were assessed using VAS scales, ranging from 0 to 10 cm (0–100 points), to evaluate physical and psychological loads, which have proven helpful in research (28). Participants were asked to rate the perceived demands of their typical workday by answering the following questions (1): Physical load: “On a typical workday, how physically demanding do you perceive your job to be?” (2); Psychological load: “On a typical workday, how mentally demanding do you perceive your job to be?”

### 2.5 Data processing and statistical analysis

All data were transferred to an Excel spreadsheet (Microsoft Excel for Mac, Version 16.85, Redmond, WA, USA). Normal distribution was verified using a combination of visual inspection and Shapiro–Wilk tests (29). Variance homogeneity was visually checked by plotting residuals and using Levene-Test. Potential between-group effects were analyzed via one-way ANOVA with post-hoc Tukey’s correction for each outcome. Furthermore, effect sizes using omega square were calculated. The level of significance was set at *p* = 0.05 for all analyses. Statistical analysis was performed with R (version 4.0.3).

## 3 Results

Characteristics of the participants are presented in Table 1. A total of 403 participants completed the interview and questionnaires, giving a response rate of 36%. About 67% of the respondents were men, and 33% were women. The majority (31%) of respondents were aged between 30 and 39 years.

**Table 1:** Characteristics of the participants.

	Age (years)	Work experience (years)	Height (cm)	Weight (kg)	BMI (kg/m^2^)
Total sample (*n* = 411)	41 ± 12	9 ± 9	173.3 ± 9.4	82.3 ± 22.9	27.08 ± 4.57
FO (*n* = 64)	41 ± 11	11 ± 10	176.3 ± 8.0	87.2 ± 14.8	28.00 ± 4.06
MA (*n* = 240)	41 ± 11	9 ± 9	174.6 ± 9.3	81.7 ± 16.3	26.73 ± 4.52
LO (*n* = 99)	42 ± 11	9 ± 10	168.4 ± 8.7	77.7 ± 16.3	27.32 ± 4.92

Mean ± standard deviation; FO = Forge; MA = Manufacturing; LO = Logistics; BMI = body mass index.

Some workers only partially completed the questionnaire; all available data were included in the analysis. 32 participants did not answer the GPAQ, one did not answer the physical and psychological load, and three did not answer the SF-36.

### 3.1 Pain frequency and intensity

308 workers (76.4%) reported at least one orthopedic issue, compared to 11% reporting at least four problems. The underlying values are presented in Table 2.

**Table 2:** Orthopedic complaints, BMI, self-reported physical activity, quality of life, physiological and psychological load of the three different working conditions.

Parameter	Forge	Manufacturing	Logistic	*p*-value	ω^2^
Orthopedic Complaints (a.u.)	45 (70%)	181 (75%)	82 (83%)		
BMI (kg/m^2^)	64 (100%) 28.0 ± 4.06	240 (100%) 26.7 ± 4.52	99 (100%) 27.3 ± 4.92	0.119	<0.01
Occupational MVPA (METs min/week)	56 (88%) 8,462 ± 5,757	239 (99%) 6,978 ± 5,137	99 (100%) 9,640 ± 4605*	<0.001	0.04
Total MVPA (METs min/week)	56 (88%) 9,243 ± 5,598	239 (99%) 8,471 ± 5,390	99 (100%) 10,856 ± 4680*	0.001	0.03
Sedentary Behavior (min/day)	56 (88%) 426 ± 152	239 (99%) 439 ± 154	99 (100%) 448 ± 155	0.709	<0.01
PCS (a.u.)	64 (100%) 47.90 ± 9.46	238 (99%) 46.68 ± 8.53	98 (99%) 45.81 ± 9.28	0.34	<0.001
MCS (a.u.)	64 (100%) 47.68 ± 9.93	238 (99%) 45.81 ± 10.12	98 (99%) 47.03 ± 10.77	0.342	<0.001
Physical load (a.u.)	64 (100%) 49.39 ± 28.41	239 (99%) 49.92 ± 24.56	99 (100%) 52.72 ± 27.39	0.618	<0.01
Psychological load (a.u)	64 (100%) 45.73 ± 29.97	239 (99%) 48.26 ± 29.97	99 (100%) 50.30 ± 29.43	0.632	<0.01

Furthermore, *F*-Test *p*-values (1 × 3 ANOVA) and effect sizes (ω^2^, omega squared) are also provided. n (%), mean ± standard deviation, BMI = body mass index (kg/m^2^), MVPA work = moderate to vigorous work activity per week (METs min/week), MVPA total = moderate-to-vigorous total physical activity per week (METs min/week), PCS = physical component summary, MCS = mental component summary, *significantly higher than manufacturing (*p* < 0.01).

**Figure 1:**
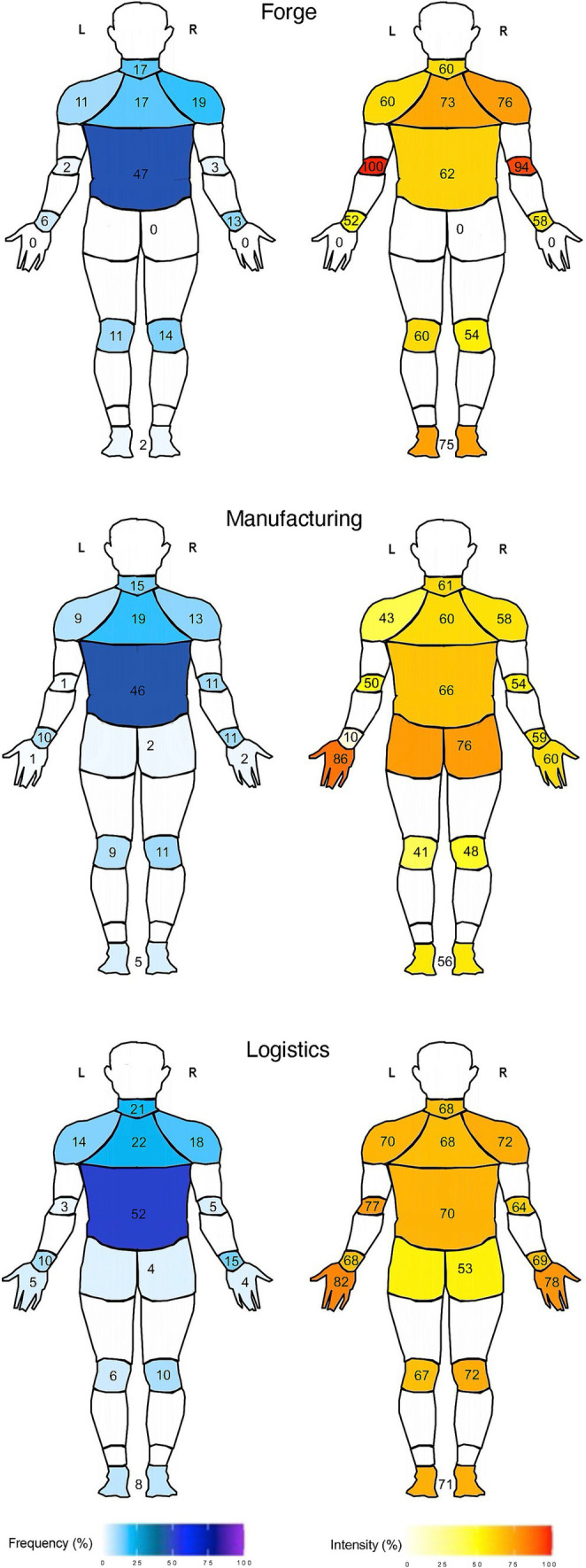
Frequency and pain-intensity of orthopedic complaints of industrial workers in the work conditions forge, manufacturing, and logistics [dorsal view (87)].

For pain intensity, only the left shoulder showed a significant effect [*F*(2,40) = 5.40; *p* = 0.008; ω^2^ = 0.17]. Post-hoc tests revealed a difference (*p* = 0.007) between the manufacturing group (*n* = 22; 42.5 ± 24.8 au) and the logistic group (*n* = 14; 70.4 ± 26.3 au), but not between the forge group and any other group. For neck [*F*(2,64) = 0.76; *p* = 0.472; ω^2^ = 0.00], upper back [*F*(2,75) = 1.89; *p* = 0.158; ω^2^ = 0.02], shoulder right [*F*(2,57) = 3.16; *p* = 0.05; ω^2^ = 0.07], elbow right [*F*(2,31) = 3.18; *p* = 0.055; ω^2^ = 0.11], elbow left [*F*(2,3) = 6.58; *p* = 0.08; ω^2^ = 0.65], wrist right [*F*(2,47) = 0.87; *p* = 0.426; ω^2^ = 0.00], wrist left [*F*(2,32) = 2.35; *p* = 0.112; ω^2^ = 0.07], low back [*F*(2,188) = 0.98; *p* = 0.378; ω^2^ = 0.00], knee right [*F*(2,42) = 3.18; *p* = 0.052; ω^2^ = 0.09], knee left [*F*(2,32) = 2.70; *p* = 0.083; ω^2^ = 0.09], foot [*F*(2,18) = 0.82; *p* = 0.457; ω^2^ = 0.00], no group effect was found. Furthermore, the orthopedic frequencies of the right hand, left hand, and hip were not represented in all groups. Therefore, no further analysis was performed. The values are shown in Figure 1.

### 3.2 Physical activity

Occupational MVPA [*F*(2,368) = 9.49; *p* < 0.001; ω^2^ = 0.04] and total MVPA [*F*(2,368) = 6.90; *p* = 0.001; ω^2^ = 0.03] showed a significant between-group difference. Post-hoc testing revealed a significant difference (*p* ≤ 0.001) between the manufacturing group (*n* = 219; 6.978 ± 5.137 METs min/week) and the logistic group (*n* = 96; 9.640 ± 4.605 METs min/week) for occupational MVPA, and between the manufacturing group (*n* = 219; 8.471 ± 5.390 METs min/week) and the logistic group (*n* = 96; 10.856 ± 4.680 METs min/week) for total MVPA. No significant group difference was found for sedentary behavior [*F*(2,368) = 0.34; *p* = 0.709; ω^2^ = 0.00].

### 3.3 Body composition

No significant group differences were detected in BMI [*F*(2,400) = 2.14; *p* = 0.119; ω^2^ < 0.00].

### 3.4 Quality of life

There were no significant group differences in MCS [*F*(2,397) = 1.08; *p* = 0.342; ω^2^ < 0.00] or PCS [*F*(2,397) = 1.08; p = 0.34; ω^2^ < 0.00].

### 3.5 Perceived load

No significant differences were observed in physical load [*F*(2,399) = 0.48; *p* = 0.618; ω^2^ < 0.00] or psychological load [*F*(2,399) = 0.46; *p* = 0.632; ω^2^ = 0.00].

## 4 Discussion

This cross-sectional study aimed to examine whether different industrial work conditions influence the health status of industrial workers. The main findings are (I) a high number of orthopedic complaints with high variability in every group and one significant difference in orthopedic complaints between the manufacturing and logistic conditions; (II) a significant difference between the manufacturing and logistic conditions on physical activity; and (III) no significant differences across all other collected data.

### 4.1 Orthopedic complaints

The literature highlights the significant burden of musculoskeletal disorders in industrial settings (30, 31), driven by various risk factors for their development (8). Our findings align with this; over 76% of the analyzed workers reported at least one orthopedic issue. This high prevalence reflects similar trends in the literature (7–9) and underscores the critical need for targeted interventions within this population. Research indicates that the interaction of biomechanical and psychosocial risk factors increases the likelihood of developing musculoskeletal disorders (32, 33).

Low back pain emerged as the most frequently reported issue among participants in our study, aligning with prior research (5, 7, 8, 10, 34). Meta-analyses revealed a mean prevalence between 37 to 51% (7–9), which is consistent with our findings. The strong correlation between workload and the prevalence of low back pain (35, 36) further highlights the need to address these factors through targeted interventions. Additionally, individual factors such as obesity, educational level, and sex have been identified as contributors to a high prevalence of musculoskeletal disorders in the lower back (7).

In addition to the lower back, a systematic review and meta-analysis identified the shoulder, neck, and wrist as the most prevalent sites for musculoskeletal disorders, with a 12-month prevalence ranging from 42 to 60% (8). Our findings align closely with these observations, highlighting similar patterns of affected body regions. In contrast, another review emphasized that the back, wrist, and elbow are the most common anatomical regions of musculoskeletal disorders (34). Furthermore, a systematic review and meta-analysis of construction workers identified the lower back, knee, shoulder, and wrist as the most affected body regions (9). Lower limb musculoskeletal disorders were reported to be less prevalent than back or upper limb, as documented in the literature (7, 8), which aligns with our findings. These variations in the literature reflect the high variability in musculoskeletal disorders, influenced by individual characteristics and work-related factors.

Notably, our study identified significant differences in pain intensity between manufacturing and logistics workers, particularly in the left shoulder. Although the specific cause for this unilateral pain remains unclear, it is plausible that task-specific physical demands or individual biomechanical factors may play a role. Other authors have recognized that there are different tasks and organizations in manufacturing than in logistics, where logistics workers must frequently bend, twist, and stand for a long period of time (37). Both conditions involve repetitive tasks with low load and high work pace (38), and physically demanding activities such as heavy lifting (38, 39). These factors are established contributors to musculoskeletal disorders (8, 31).

However, these findings underscore the complexity of addressing musculoskeletal disorders in industrial settings, where workers’ tasks and conditions can differ significantly. Therefore, the observed pain in the left shoulder might be attributed to a combination of individual and job-specific factors. The high variability in orthopedic complaints and adverse working conditions pose challenges to the development of generalized interventions. As a result, translating findings into effective solutions requires a focus on tailored strategies that consider individual worker characteristics and specific job demands (40, 41).

Beyond the physical health implications, musculoskeletal disorders affect workability (6, 32), particularly in the low back area (42), prolonged absences (8), and substantial financial costs (39). They are also one of the leading causes of permanent incapacity (43), productivity loss, and early retirement (11).

Interventions such as ergonomic adaptations and innovative technologies, including robots and exoskeletons (44), might help prevent work-related musculoskeletal disorders by alleviating the physical strain associated with industrial tasks. These approaches are particularly relevant in countries experiencing demographic shifts that challenge the sustainability of physically demanding jobs (3) and human decline in musculoskeletal mass, leading to reduced adaptation strategies (7).

### 4.2 Body composition

A higher prevalence of overweight and obesity among industrial workers than among the general population is known (30). With a BMI of approximately 27 kg/m^2^, our findings fall within the WHO classification of overweight (45). However, we did not find a significant difference between the conditions, but there was a high variance in the data.

Generally, an increased BMI and musculoskeletal disorders are associated with each other (7), and both negatively impact work-related outcomes (30). Additionally, obesity is related to musculoskeletal pain (46). Furthermore, obesity with fat depots is recognized as a significant pro-inflammatory factor in modern society that contributes to modern diseases such as cancer, metabolic disorders, cardiovascular diseases, and dementia (47). Prevention and, if reasonable, therapy are necessary to improve health and increase healthspan, ideally targeting multiple health factors such as musculoskeletal disorders and obesity (48).

### 4.3 Physical activity

Promoting physical activity in the workplace has been a well-established health strategy for decades (49). According to prevailing guidelines, optimal physical activity is at least 600 METs min/week (50). Individuals falling below this WHO recommendations threshold may be classified as physically inactive (22). In our study, the manufacturing group exhibited the lowest level of occupational MVPA, at 6.978 METs min/week; only four individuals were labeled as physically inactive. However, self-reported data may lead to overestimation of physical activity, particularly in urban areas (51). In addition, participants appeared to overestimate their MVPA and underestimate their sedentary behavior when using the GPAQ, suggesting that the results should be interpreted with caution (24).

Occupational physical activity exceeds the recommended threshold 11-fold and can be considered a physical health paradox (52–54). While the positive association between leisure-time physical activity, orthopedic issues, and cardiovascular disease mortality is well documented (53), occupational physical activity did not have a beneficial association with mortality or orthopedic complaints (53, 54). On the contrary, high levels of occupational physical activity increase the risk for adverse health outcomes, mortality, and orthopedic complaints (53, 54). Consequently, promoting decreased physical activity among industrial workers could improve workplace health. While leisure-time physical activity is important for overall health (55), our study found that non-occupational activity accounted for only a modest difference in total physical activity. Given the potential health risks of occupational physical activity, it is essential to consider individual lifestyle factors.

Regarding sedentary behavior, no differences were observed across work conditions, which ranged between 426 and 448 min. Despite high physical activity levels, participants sit for over 7.5 h daily, exceeding the recommended limit for high sedentary behavior (56). However, sitting for long periods may be a relevant health factor, including posture during sitting from an evolutionary perspective (57).

A notable difference in the manufacturing and logistics groups was observed for both occupational MVPA and total MVPA, with differences of over 660 min and approximately 600 min, respectively. To contextualize these differences, the WHO recommends at least 150 to 300 min of moderate physical activity per week (58). However, the data showed that job profiles and individual lifestyle factors must be considered when planning and implementing workplace health promotion, especially for those with high physical activity.

### 4.4 Quality of life

Previous research has established a positive correlation between factors such as workability, nutritional intake, and sleep quality on quality of life (59–61). While these studies highlighted the influence of various factors on quality of life, our study found no significant differences across work conditions. However, our mental and physical scores were lower than those found in other studies with comparable populations (59), and similar to the data from Lim and colleagues (61) for night-shift workers, who comprised most of our participants.

Moreover, our quality of life scores were lower in terms of MCS (50.04) than those of patients with low back pain or disc herniation but higher in PCS (44.51) (62). Compared to adults in Germany, our results were lower in both categories (MCS: 51.40; PCS:49.30) (63). Orthopedic complaints could contribute to PCS scores across a range of patients with low back pain to general adults in Germany, but this remains speculative.

Of all participants, 56% had a mental and physical score below 50, which matches the percentage reported by Ghasemi and colleagues (64) (59%). Others have found that one-third of construction workers experience a mental health condition, resulting in high losses in work time and high economic costs (65). The number of sick days taken due to mental health concerns in the workplace has increased, which is in line with the rising trend of mental illnesses (66, 67). In particular, shift workers are affected by this trend, with a higher prevalence of poor mental health, particularly depressive symptoms (68, 69). Workers’ exposure to psychosocial hazards is influenced by the interplay between job demands and resources (70), whereas job control may be a possible influencing factor in the manufacturing context (40).

### 4.5 Practical applications

This study offers valuable insights and practical implications for workplace health promotion. Our analysis confirmed the diversity of job profiles among industrial workers. The nature of these job profiles is influenced by factors such as work environment, activities, and human factors (71, 72). While ergonomic concerns, particularly orthopedic issues, have historically been the focus of workplace health initiatives, our findings underscore the critical need to address psychosocial factors (35). Unpredictable work hours, for example, hinder workers’ access to medical care, contributing to undetected health conditions, poor overall health, and an increased risk of workplace injuries (73). These challenges negatively affect worker safety and contribute to organizational issues, such as productivity, absenteeism, and rising healthcare costs (14).

Companies strive to meet the increasing expectations of their workers by implementing progressively more comprehensive measures to address these demands (74). This reinforces the importance of aligning health promotion strategies with specific needs and expectations of the workforce. In this regard, recent reviews highlighted that workplace interventions, particularly in high-risk industries, are associated with a measurable reduction in musculoskeletal disorders (31) and stress-related absenteeism (68). Studies have demonstrated that health promotion programs reduce the prevalence of physical ailments like low back pain and alleviate mental stress, contributing to a healthier workforce (32, 75). Besides ergonomics, education is essential and plays a vital role in managing health by equipping workers with skills to adopt a healthier behavior (76).

To address these challenges, practical interventions should adopt a dual approach that combines preventive and rehabilitative strategies tailored to the unique worker and individual and job-specific needs. Strategies such as structured duty schedules, modified working postures, job rotation strategies, and targeted training programs are required to manage workplace health issues effectively (3, 77, 78). Given the substantial variability in job demands and individual health conditions, frameworks such as the Goldilocks principle (79), which seeks to balance workload demands, and the IGLO framework (80), which targets health promotion at the individual, group, leader, and organizational levels, offer valuable guidance in this regard.

Technological advances can further enhance these strategies. For instance, advanced monitoring technologies, such as wearable devices, allow real-time monitoring of individual health status (81), provide precise data (82), and enable tailored intervention recommendations (83). Moreover, after identifying specific job profiles and individual health conditions, e-health platforms offer a promising solution for delivering accessible interventions that accommodate irregular work among industrial shift workers (84). These tools can facilitate personalized health management, improve resource accessibility, and foster proactive health behaviors (85).

Future workplace interventions should systematically integrate these frameworks and technologies to classify health states and develop tailored health-management strategies. For example, task rotation schemes (86) customized with individual psychosocial support (67) can help workers meet their roles’ physical and mental demands. The cross-sector applications of such interventions could further validate their effectiveness and adaptability across various industries.

### 4.6 Limitations

Nonetheless, this study had several limitations. A cross-sectional design restricts the ability to infer causality from observed relationships between work conditions and health outcomes. Furthermore, the participants were drawn from a single mid-sized steel company, which restricts the generalizability of our findings to other industrial sectors and broader occupational populations. Potential confounding variables, such as age, sex, and lifestyle habits, were not comprehensively accounted for and may influence the observed relationships. Additionally, the response rate introduces a possible bias that could affect the validity of the findings.

Another notable limitation is the lack of longitudinal data, which prevents the tracking of health outcomes and evaluating their progression. Future research should prioritize longitudinal designs to assess the durability of health improvements and their influence on organizational outcomes. Moreover, the literature has reported a higher prevalence of musculoskeletal disorders in certain body regions when specific tools are used (8). This highlights the potential variability in reported outcomes based on the methodology employed. Similarly, reliance on self-defined musculoskeletal disorders has been associated with higher incidence rates (7), indicating the need for standardized definitions and assessments in future studies.

In terms of methodology, while we utilized personal interviews to address the inherent challenges of self-reported data, this approach remains subject to recall and reporting bias. Complementing self-reported measures with objective health data or workplace observations in future studies could enhance the reliability of findings.

For broader applicability, systematic reviews comparing interventions across different cultural and regulatory settings could provide valuable insights into a global adaptation of workplace health strategies. Future research should explore how parameters at the individual level, such as physical activity, impact health-related outcomes (55), particularly under more homogenous or task-specific work conditions. This highlights the potential impact of such interventions, particularly in shaping specific job profiles and addressing work-related health issues. Additionally, emphasis should be placed on fostering adequate work conditions within a broader health-related system and ensuring the sustainability and scalability of these approaches.

## 5 Conclusion

This study provides valuable insights into working conditions and health status of industrial shift workers. We observed a high prevalence of orthopedic complaints, low quality of life scores, and significant differences in physical activity across work conditions. However, the variability within each work condition suggests that more than stratifying workers based solely on work conditions may be required for effective workplace health interventions. While clear distinctions between work conditions and health outcomes remain challenging, our findings emphasize the importance for a comprehensive approach to workplace health promotion. Successful preventive and rehabilitative programs should focus on an individual level by implementing job-specific profiles and regular health assessments, including adequate screening and monitoring procedures among industrial shift workers. This approach can potentially improve workers’ health, enhance productivity, and support a more sustainable workforce.

## Data Availability

The raw data supporting the conclusions of this article will be made available by the authors, without undue reservation.
